# The Medicinal Timber *Canarium patentinervium* Miq. (Burseraceae Kunth.) Is an Anti-Inflammatory Bioresource of Dual Inhibitors of Cyclooxygenase (COX) and 5-Lipoxygenase (5-LOX)

**DOI:** 10.5402/2013/986361

**Published:** 2013-10-01

**Authors:** R. Mogana, K. Teng-Jin, C. Wiart

**Affiliations:** Center for Natural and Medicinal Products Research, School of Pharmacy, Faculty of Science, University of Nottingham (Malaysia Campus), Jln Broga, 43500 Semenyih, Selangor Darul Ehsan, Malaysia

## Abstract

The barks and leaves extracts of *Canarium patentinervium* Miq. (Burseraceae Kunth.) were investigated for cyclooxygenase (COX) and 5-lipoxygenase (LOX) inhibition via *in vitro* models. The corresponding antioxidative power of the plant extract was also tested via nonenzyme and enzyme *in vitro* assays. The ethanolic extract of leaves inhibited the enzymatic activity of 5-LOX, COX-1, and COX-2 with IC_50_ equal to 49.66 ± 0.02 *μ*g/mL, 0.60 ± 0.01 *μ*g/mL, and 1.07 ± 0.01 *μ*g/mL, respectively, with selective COX-2 activity noted in ethanolic extract of barks with COX-1/COX-2 ratio of 1.22. The ethanol extract of barks confronted oxidation in the ABTS, DPPH, and FRAP assay with EC_50_ values equal to 0.93 ± 0.01 *μ*g/mL, 2.33 ± 0.02 *μ*g/mL, and 67.00 ± 0.32 *μ*g/mL, respectively, while the ethanol extract of leaves confronted oxidation in *β*-carotene bleaching assay and superoxide dismutase (SOD) assay with EC_50_ value of 6.04 ± 0.02 *μ*g/mL and IC_50_ value of 3.05 ± 0.01 *μ*g/mL. The ethanol extract acts as a dual inhibitor of LOX and COX enzymes with potent antioxidant capacity. The clinical significance of these data is quite clear that they support a role for *Canarium patentinervium* Miq. (Burseraceae Kunth.) as a source of lead compounds in the management of inflammatory diseases.

## 1. Introduction

Inflammation is a physiological response process that is generated by the body in the event of oxidative stress, injury, infection, or irritation. Chronic inflammation involves the release of a number of mediators, resulting in the proliferation of fibroblasts and vascular endothelium, as well as lymphocytes, plasma cells, and macrophages [1]. The release of all these mediators can contribute to chronic degenerative diseases such as arthritis, cancer, heart disease, Alzheimer's disease, diabetes, and asthma, which may increase disease-associated morbidity. Inflammation in injured cells is both initiated and maintained by the overproduction of prostaglandins and leukotrienes, which are produced by separate enzymatic pathways, namely, the cyclooxygenase (COX) and lipoxygenase (LOX) pathways, respectively. Both the prostaglandins as well as the leukotrienes are on demand biosynthesised from arachidonic acid (AA), which is a 20-carbon fatty acid, derived from the breakdown of cell membrane phospholipids by any number of phospholipase A2 (PLA_2_) isoforms. AA is then further metabolized by the COX and 5-LOX enzyme systems to a variety of mediator molecules, including prostaglandin (PG) E_2_, thromboxanes (TXs) (TXA_2_), prostacyclins (PGI_2_), and highly inflammatory leukotrienes such as leukotriene (LT) B_4_, LTC_4_, and LTD_4_.

Conventional pharmacological management of inflammatory disease like osteoarthritis involves treatment with nonsteroidal anti-inflammatory drugs (NSAIDs) or selective COX-2 inhibitors that block the formation of PGs without modulating 5-LOX enzyme activity. Inhibition of one or both of the COX enzymes may “shunt” AA metabolism down the 5-LOX pathway, which can aggravate toxicity associated with the lack of PGs and excess production of LTs. For example, NSAID-induced gastric ulcers have been shown to have high concentrations of LTB_4_ in their walls, which attract leukocytes to the stomach and may contribute to ulceration [2, 3].

Recently, reports have appeared regarding so-called “dual inhibitors,” agents that inhibit not only COX-1 and COX-2 but also 5-LOX [4–8]. These agents with antioxidative properties may be particularly effective for managing the metabolic processes underlying inflammatory conditions and reducing both gastric and cardiovascular side effects by balancing AA metabolism in the body.

In continuation of our earlier studies on the pharmacological properties of* Canarium patentinervium* Miq. that established its *in vitro* antibacterial, antioxidant, and antitumor activity [9–11], this study investigates the inhibition of 5-LOX, COX-I, and COX-2 and the total antioxidant capacity of *Canarium patentinervium* Miq. *Canarium patentinervium* Miq. is a rare medicinal timber from the genus *Canarium* and family of Burseraceae found in Asia Pacific region previously recorded for its usage in wound healing by the indigenous people [11, 12]. The above dual inhibition study by our team is the first documented study reported on this plant.

## 2. Materials and Methods

### 2.1. Plant Material

The leaves and barks of *Canarium patentinervium* Miq. were collected from one individual tree from Bukit Putih, Selangor, Malaysia (3°5′24′′N 101°46′0′′E). The plant was identified by Mr. Kamaruddin (Forest Research Institute of Malaysia). A herbarium sample (PID 251210-12) has been deposited in the Forest Research Institute of Malaysia. The leaves were air-dried and grinded into small particles using an industrial grinder. 

### 2.2. Chemical and Reagents

1,1-diphenyl-2-picryhydrazyl (DPPH), Trolox (6-hydroxy-2,5,7,8-tetramethylchromon-2-carboxylic acid), 2,2-azino-bis(3-thylbenzothiazoline-6-sulfonate), 2,4,6-tripyridyl-s-triazine (TPTZ), quercetin, gallic acid, *β*-carotene, 5,5′-Dithio-bis(2-nitrobenzoic) acid (DTNB), galantamine, nordihydroguaiaretic acid (NDGA), Folin-Ciocalteu reagent, bovine hemin chloride, *N,N,N*′*,N*′-tetramethyl-p-phenylenediamine dihydrochloride (TMPD), Tris-CL, and SOD kit were purchased from Sigma Aldrich. Sodium chloride, ascorbic acid, ferric chloride, and glacial acetic acid were purchased from System. Hexane and chloroform were purchased from Friendemann Schmidt Chemicals. Methanol and ethanol 95%, potassium persulfate powder, ferric chloride, ferrous sulphate, Tween 20, and potassium phosphate were purchased from Kollin Chemicals. DMSO was from R & M Marketing, Essex UK. Linoleic acid, AA, superoxide dismutase (SOD), COX-1, and COX-2 were purchased from Cayman Chemical Company. Enzyme 5-lipoxygenase enzyme (human recombinant) was purchased from Calbiochem.

### 2.3. Extraction

Dried and grinded sample of leaves (2.8 kg) were soaked in hexane with the ratio of 1 : 3 parts (w/v) of sample to solvent for 2 h in a 60°C water bath and then filtered and concentrated with a rotary evaporator (Buchi, R-200 Switzerland). This was repeated three times. Thereafter the leaves and barks were left to air dry completely for three days before repeating the whole process with chloroform and then ethanol, respectively. Extracts were kept at −20°C until further use. The extracts were indicated as LH (leaf hexane extract), LC (leaf chloroform extract), LE (leaf ethanol extract), BH (bark hexane extract), BC (bark chloroform extract), and BE (bark ethanol extract), respectively.

### 2.4. Determination of Total Phenolic and Total Flavonoid of Extracts

The total phenolic content of the extracts was determined with the Folin-Ciocalteu reagent, following the modified method of Singleton and Rossi [13]. Briefly, 20 *μ*L of test samples dissolved in methanol (1 mg/mL) was added to 100 *μ*L of Folin-Ciocalteu reagent and 1.58 mL of deionised water. After allowing the mixture to stand at room temperature for 5 min, 300 *μ*L of 20% (w/v) sodium carbonate was added. Reaction mixtures were further incubated at room temperature for 30 mins, following which absorbance at 765 nm was read against a blank, using a Jenway 6305 UV-Vis spectrophotometer (Jenway Ltd., Essex, UK). The standard calibration curve was plotted using gallic acid (50–250 *μ*g/mL), from which total phenolic content was expressed as gallic acid equivalents (mg/g extract). The total flavonoid content of the crude extract was determined with the aluminium chloride colorimetric method of Froehlicher et al. [14]. Briefly, 0.5 mL of test samples dissolved in methanol (1 mg/mL) was added to 1.5 mL of 2% methanolic solution of aluminium chloride in sealed tubes and kept in dark for 15 mins. Absorbance was then read at 430 nm using UV-vis spectrophotometer against blank of methanolic aluminium chloride solution. The standard calibration curve was plotted using quercetin (50–250 *μ*g/mL), from which total flavonoid content was expressed as quercetin equivalents (mg/g extract).

### 2.5. Antioxidant Capacity Tests

Aliquots of extracts were dissolved in dimethyl sulfoxide (DMSO, R & M) prior to assay. Trolox, vitamin C (l-ascorbic acid), and quercetin were used as positive control. The positive controls were indicated as AA (ascorbic acid), QC (quercetin), TRO (trolox), and SOD (superoxide dismutase enzyme), respectively. Assay was performed using Thermo Scientific Varioskan Flash microtiter plate reader, linked to a computer equipped with (SkanIt Software 2.4.3). EC_50_ (concentration of a compound where 50% of its maximal effect is observed) values were determined using Prism 5.00 software. At least three independent tests were performed for each sample. All results were calculated after correcting for colour absorbance of the plant extract as sample blank. 

#### 2.5.1. 2,2-Azino-bis(3-thylbenzothiazoline-6-sulfonate) (ABTS) Assay

The ABTS assay, as described by Miller et al. [15], Rice-Evans [16], and Re et al. [17], was employed to determine the radical scavenging activity of the plant extracts. ABTS assay involves the reduction of the blue-green 2,2-azino-bis(3-thylbenzothiazoline-6-sulfonate) radical cation (ABTS^●+^) by antioxidants to its original colourless ABTS form. Greater discolouration results in lower absorbance at 734 nm indicating higher antioxidant capacity [15, 16]. Samples were plated out in triplicate in 96-well microtiter plate at different concentrations. Trolox, vitamin C (l-ascorbic acid), and quercetin were used as positive control which was prepared in ethanol, and serial dilutions of this positive control were also prepared. Ethanol was used as the negative control. The stock solution included 7 mM ABTS solution and 2.4 mM potassium persulfate solution. The working solution was then prepared by mixing the two stock solutions in equal quantities. This solution was then stored in the dark for 12–16 hours in order to stabilise it before use. It remains stable for two to three days in the dark. The concentrated ABTS+ solution was diluted with cold ethanol shortly before conducting the assay, to a final absorbance of 0.70 ± 0.01 at 734 nm at 37°C, in a 3 cm cuvette. The total scavenging capacity of the extracts was quantified through the addition of 100 *μ*L ABTS+ to 100 *μ*L of test sample. The solutions were heated to 37°C for 7 min, after which the absorbance was read at 734 nm. The percentage decolouration was calculated using the following equation, and the extent of inhibition of the absorbance of the ABTS+ was plotted as a function of the concentration. This activity is given as percent ABTS radical scavenging, which is calculated with the equation: ABTS radical scavenging capacity (%) = [(*A*_control_ − *A*_sample_)/(*A*_control_)] × 100, where *A*_control_ is the absorbance of ABTS radical + ethanol; *A*_sample_ is the absorbance of ABTS radical + sample extract/standard. Sample well with ethanol devoid of ABTS was used as ABTS blank control to correct colour absorbance of samples.

#### 2.5.2. Ferric Reducing Ability of Plasma (FRAP) Assay

The antioxidant activity samples were determined using the colorimetric FRAP assay, as described by Benzie and Strain [18] with slight modifications. FRAP is the ferric reducing power of antioxidants by the reduction of the ferric ions to the ferrous ions, which form a blue colored ferrous-tripyridyltriazine complex (ferric TPTZ) which is detected at 593 nm. Deeper blue colour indicates higher antioxidant potential [19]. Aliquots of samples were plated out in triplicate in a 96-well microtiter plate at different concentrations. The working FRAP reagent was prepared just before assay by mixing 300 mM of acetate buffer (pH 3.6), 10 mM of TPTZ, and 20 mM of FeCl_3_·6H_2_O in ratio of 10 : 1 : 1. Briefly 180 *μ*L of the FRAP reagent was mixed with 20 *μ*L of the test sample so that the final dilution of the test sample in the reaction mixture was 1/10. After 30 minutes, the absorbance of the coloured product (ferrous tripyridyltriazine complex) was recorded. Fe (II) concentrations in the range of 1 *μ*M–100 *μ*M (FeSO_4_·7H_2_O) were used as standard for calibration curve, and equation of linearity is determined (*y* = *ax* + *b*). From the linearity equation, concentration of sample that produced the same absorbance as 1 mM of Fe (II) was determined (*y* of sample filled in equation to obtain *x*). The antioxidant activity was calculated as ferrous equivalents, the concentration of samples which produced an absorbance value equal to that of 1 mM FeSO_4_. Sample well without FRAP reagent was used as sample blank control to correct colour absorbance of samples.

#### 2.5.3. *β*-Carotene Bleaching Assay

The *β*-carotene bleaching assay was conducted according to the method described by Habtemariam and Jackson [20] with some modifications. In the *β*-carotene/linoleic model, linoleic acid reacts with ROS (reactive oxygen species, i.e., chemically reactive molecules containing oxygen) and O_2_ to form an unstable peroxy radical. *β*-carotene being an antioxidant will react with this radical to form stable epoxide causing the bleaching of yellow solution. Competition reaction occurs with the presence of another antioxidant (sample) to react with the peroxy radical resulting in slower bleaching of solution detected at 470 nm spectrophotometrically [21]. Aliquots of samples were plated out in triplicate in a 96-well microtiter plate at different concentrations. Briefly, 1 mL of a *β*-carotene solution in chloroform (2 mg in 10 mL) was pipetted into a round bottom flask containing 40 *μ*L of linoleic acid and 500 *μ*L of Tween 20. After the removal of chloroform using a rotary vacuum evaporator at 45°C, 100 mL of deionised water was added with vigorous agitation. 180 *μ*L of the emulsion was added to 20 *μ*L of test samples at varying concentrations in 96-well microtitre plate. The absorbance was measured at 470 nm immediately against a blank consisting of the emulsion without *β*-carotene and after 3 h of incubation at 50°C using a spectrophotometer. The antioxidant activity of test agents was evaluated in terms of bleaching of *β*-carotene using the following formula: Antioxidant activity AA (%) = [1 − (*A*_0_ − *A*_*t*_)/(*A*_0_′ − *A*_*t*_′)] × 100, where *A*_0_ and *A*_0_′ are absorbances measured at zero time of incubation for the test sample and control, respectively; *A*_*t*_ and *A*_*t*_′ are the absorbances measured in the test sample and control, respectively, after incubation for 3 h. Sample well without *β*-carotene was used as sample blank control to correct colour absorbance of samples.

#### 2.5.4. Superoxide Dismutase (SOD) Assay

This assay was conducted with Sigma SOD assay kit [22]. SOD Assay Kit-WST allows very convenient SOD assaying by utilizing Dojindo's highly water-soluble tetrazolium salt, WST-1 (2-(4-Iodophenyl)-3-(4-nitrophenyl)-5-(2,4-disulfophenyl)-2H-tetrazolium, monosodium salt), that produces a water-soluble formazan dye upon reduction with a superoxide anion. In this study the enzymatic system that uses xanthine oxidase (XOD) to generate O_2_^−^ radicals was used [23]. XOD was used for formation of O_2_^−^ that is reduced to oxygen by SOD-like samples. The WST-1 is used as a probe that undergoes competitive reaction with SOD-like samples to reduce O_2_^−^ to oxygen forming a formazan coloured dye. The rate of reduction with oxygen is linearly related to the xanthine oxidase (XO) activity and is inhibited by SOD. Therefore, the IC_50_ (50% inhibition activity of SOD or SOD-like materials) can be determined by a colorimetric method. Since the absorbance at 450 nm is proportional to the amount of superoxide anion, the SOD activity as an inhibition activity can be quantified by measuring the decrease in the colour development at 450 nm. Twenty microliters of sample were plated at different concentrations in a 96-well microtiter plate. Then, 200 *μ*L of WST solution and 20 *μ*L of enzyme were added and incubated for 20 mins at 37°C. Absorbance was then recorded at 450 nm using Thermo Scientific Varioskan Flash microtiter plate reader, linked to a computer equipped with (SkanIt Software 2.4.3). The SOD inhibition rate and IC_50_ (concentration of a compound that is required for 50% inhibition *in vitro*) were determined. Superoxide dismutase enzyme (Nacalai Tesque) was used as standard control. Sample well with buffer was used as sample blank control to correct colour absorbance of samples.

### 2.6. The 5-LOX Inhibition Assay

The 5-lipoxygenase assay was conducted according to the method described by Baylac and Racine [24] and Kamatou et al. [25] with some modifications. 5-lipoxygenase enzyme (human recombinant from Calbiochem) was used. Ice-cold buffer (potassium phosphate) at 4°C was mixed with 100 U of the thawed enzyme. Twenty microliters of samples dissolved in DMSO were plated out in triplicate in a 96-well microtiter plate at different concentrations, followed by 160 *μ*L of 0.1 M potassium phosphate buffer (pH 6.3) and maintained at 25°C and 20 *μ*L of enzyme solution. Mixture was agitated, and 10 *μ*L of linoleic acid was added and incubated for 10 mins at 25°C. Absorbance was recorded at 234 nm using Thermo Scientific Varioskan Flash microtiter plate reader, linked to a computer equipped with (SkanIt Software 2.4.3). 5-Lipoxygenase is known to catalyse oxidation of unsaturated fatty acids containing 1-4 diene, and the modification of linoleic acid (1-4-diene into 1-3-diene) can be detected at 234 nm. Percentage inhibition of enzyme was determined by comparison of rates of reaction of samples relative to blank sample using the formula: (*E* − *S*)/*E* × 100, where *E* is the activity of enzyme without test sample and *S*  is the activity of enzyme with test sample. The experiments were done in triplicate. Nordihydroguaiaretic acid (NDGA) was used as positive control. Sample well with buffer was used as sample blank control to correct colour absorbance of samples.

### 2.7. The Peroxidase Endpoint Assay for COX-1 and COX-2

The COX-1 and COX-2 peroxidase end-point assay was conducted according to the method described by Gierse and Koboldt [26]. COX-1 and COX-2 enzyme from Cayman were used. In this study, COX activity is measured by utilizing peroxidase activity and the electron donor TMPD, which turns blue upon reduction as a cosubstrate. Arachidonic acid is used as a substrate which must first be converted to hydroperoxide, thus yielding an indirect measure of COX activity. This assay has been noted to be a high throughput method [26]. Twenty microliters of sample dissolved in DMSO were plated in triplicates at different concentrations in a 96-well microtiter plate followed by 20 *μ*L of 10 U/mL of enzyme solution. Then, 160 *μ*L of endpoint assay mix consisting of 100 *μ*M bovine hemin chloride, 10 mM of AA, 17 mM of TMPD, and 1 M of buffer Tris-Cl was added and incubated for 10 mins at 25°C. Absorbance was recorded at 611 nm using Thermo Scientific Varioskan Flash microtiter plate reader, linked to a computer equipped with (SkanIt Software 2.4.3). Percentage inhibition of enzyme was determined by comparison of rates of reaction of samples relative to blank sample using the formula: (*E* − *S*)/*E* × 100, where *E* is the activity of enzyme without test sample and *S*  is the activity of enzyme with test sample. Indomethacin (INDO) was used as positive control. Sample well with buffer was used as sample blank control to correct colour absorbance of samples.

### 2.8. Statistical Analysis

Concentration-response curves were calculated using the Prism software package 5.00 for Windows, GraphPad Software, San Diego, California, USA, http://www.graphpad.com/ (GraphPad, San Diego, USA), and data were obtained from three independent experiments, each performed in triplicates (*n* = 9) and represented as mean ± SD. Nonlinear best fit was plotted with mean ± SD. One-way ANOVA was performed followed by Tukey's multiple comparison tests. Throughout the analysis, *P* < 0.05 was considered significant.

## 3. Results and Discussions

### 3.1. Extraction Yield, Total Phenolic, and Flavonoid Contents

Table 1 presents the yield and the total phenolic and flavonoid contents of the extracts. The ethanol extract of leaves had the highest yield amongst the six extracts. In the Folin-Ciocalteu assay, gallic acid was used as a standard (*y* = 0.1762*x* + 0.0047, *r* ^2^ = 0.9994), and the results were expressed in gallic acid equivalent (mg/g). The total phenolic contents of the six extracts varied from 6.61 to 204.97 mg/g extract. The total flavonoid content was evaluated using quercetin as a standard (*y* = 0.0093*x* − 0.00231; *r* ^2^ = 0.9992, with *x* as the concentration of quercetin in mg/mL). Our study revealed the presence of high amount of phenolic compounds and flavonoid in the ethanol extracts of leaves with value of 204.97 mg/g GAE and 125.32 mg/g quercetin equivalent, respectively.

### 3.2. Antioxidant Capacity

*In vitro* antioxidant capacity can be determined by hydrogen atom transfer (HAT) method and single electron transfer (SET) method [27]. HAT-based methods measure the ability of an antioxidant to scavenge free radical by hydrogen donation to form a stable compound. SET-based methods detect the ability of the antioxidant to transfer one electron to reduce compound including metals, carbonyls, and radicals [28, 29]. *β*-carotene bleaching assay involves HAT method, and FRAP assay involves SET method, while DPPH and ABTS assay involve both methods predominantly via SET method [23, 30].

Table 2 presents the EC_50_ values for the respective assays. In the FRAP assay, the samples absorbance equivalent to 1 mM FeSO_4_ was calculated from equation of linearity of individual sample. Total FRAP value was determined from the absorbance obtained from the samples above using the standard Fe (II) calibration curve equation (*y* = 0.0105*x* + 0.0136, *r* ^2^ = 0.9817). Ethanol extract of barks displayed significant (*P* < 0.05) FRAP value (67.00 ± 0.32 *μ*g/mL) compared to ascorbic acid (347.00 ± 0.23 *μ*g/mL) and quercetin (86.00 ± 0.24 *μ*g/mL). In the *β*-carotene bleaching assay, ethanol extract of leaves and barks displayed the best EC_50_ value of 6.04 ± 0.02 *μ*g/mL and 7.04 ± 0.04 *μ*g/mL, respectively.

In the ABTS assay, ethanol extract of the bark displayed significant (*P* < 0.05) EC_50_(0.93 ± 0.01 *μ*g/mL) compared to ascorbic acid (1.54 ± 0.06 *μ*g/mL). In our previous study of DPPH assay (another SET method), ethanol extract of leaves and barks displayed significantly lower EC_50_ value of 2.93 ± 0.00 *μ*g/mL and 2.33 ± 0.02 *μ*g/mL, respectively, compared to trolox (EC_50_ = 4.77 ± 0.04 *μ*g/mL) [11]. The DPPH and ABTS assays have the same mechanism of action but, in most cases, the results obtained from the ABTS assay are higher than those from DPPH assay. It has been documented that results reported for the ABTS assay do not only take into account the activity of the parent compound but also the contribution of reaction products and other individual compounds on the activity, which is not the case in the DPPH assay [31, 32].

Superoxide dismutase (SOD), which catalyses the dismutation of the superoxide anion (O_2_^−^) into hydrogen peroxide and molecular oxygen, is one of the most important antioxidative enzymes. Preventive antioxidants, such as SOD are described either as preventing introduction of initiating radicals or as inhibiting the rate where new chains are set up. The ethanol extract of the leaves shows the highest SOD-like activity with IC_50_ of 3.05 ± 0.01 *μ*g/mL.

In the above experiments, the ethanol extract of leaves and barks displayed superior antioxidant capacities. The EC_50_ values of the samples were consistently low in SET methods (ABTS, DPPH, and FRAP) superior to standard as opposed to HAT method (*β*-carotene bleaching) assay. The present study demonstrates that *Canarium patentinervium* Miq. is a potent source of antioxidants that exhibits its antioxidant activity predominantly via the SET method.

Several reports emphasized on the fact that there is a positive relationship between total phenolic contents and antioxidant activity [33, 34]. Our study demonstrated the existence of a low positive correlation between the total phenolic contents and the FRAP values (*y* = −0.0125*x* + 2.2564, *r* ^2^ = 0.5246) and total phenolic contents and *β*-carotene bleaching values (*y* = −0.2747*x* + 52.42, *r* ^2^ = 0.2287), respectively. Therefore, one could draw an inference that nonphenolic compounds by itself or in synergy with the phenolics and flavonoids impart *Canarium patentinervium* Miq. its antioxidant properties.

### 3.3. Anti-Inflammatory Activity

This assay measures the inhibitory activity against 5-LOX and COX enzymes. At the onset of the inflammatory process, arachidonic acid is converted to eicosanoids and leukotriene B_4_ (LTB_4_) by LOX [35], which is coupled with the production of prostaglandins and thromboxanes by cyclooxygenase (COX). These inflammatory mediators are responsible for the powerful chemoattractive effects on the eosinophils, neutrophils, and macrophages, as well as the increased release of proinflammatory cytokines by macrophages and lymphocytes. 

In the 5-LOX assay, chloroform extract of the barks displayed potent enzyme inhibition (IC_50_ = 29.53 ± 0.03 *μ*g/mL) which was relatively similar to nordihydroguaiaretic acid (IC_50_ = 29.19 ± 0.02 *μ*g/mL). Ethanol extract of leaves and barks follows through with IC_50_ = 49.66 ± 0.02 *μ*g/mL and IC_50_ = 59.06 ± 0.07 *μ*g/mL, respectively. Thus the potency of activity against 5-LOX was BC > LE > BE (Table 3). According to the 5-LOX enzyme inhibition activity measurement guide by Kamatou et al. [25] and Paraskeva et al. [32] (IC_50_ < 30 *μ*g/mL: good activity; 30 < IC_50_ < 80 *μ*g/mL: moderate activity; IC_50_ > 80 *μ*g/mL: poor activity), chloroform extract of barks of *Canarium patentinervium* Miq. displays good 5-LOX enzyme inhibition activity followed by moderate activity by ethanol extract of leaves and barks.

COX (prostaglandin H_2_ synthase) inhibition is the mechanism of action of most NSAIDS. COX-1 is a constitutive form of the enzyme that has been linked to the production of physiologically important prostaglandins that may play a role in homeostasis (gastric, renal, etc.). COX-2 is a form of the enzyme that is inducible by cytokines and growth factors. Induction of COX-2 is linked to inflammatory cell types and tissues. There is an ongoing effort to identify compounds that might inhibit COX-2 in preference to COX-1 since such an agent may be safer and perhaps more efficacious. In the COX assay, only ethanol extract of leaves and barks exhibited IC_50_ below 100 *μ*g/mL. Ethanol extract of leaves had superior COX-1 inhibition (IC_50_ = 0.60 ± 0.01 *μ*g/mL) compared to COX-2 inhibition (IC_50_ = 1.07 ± 0.01 *μ*g/mL), whereas the barks had superior COX-2 inhibition (IC_50_ = 9.39 ± 0.03 *μ*g/mL) as opposed to COX-1 (IC_50_ = 11.41 ± 0.03 *μ*g/mL). According to Burnett et al. [36], Yang et al. [37], and Jim and Mark [38], a COX-1 selective inhibitor will have ratio <1, whereas a COX-2 selective inhibitor will have ratio of >1. In this study ethanol extract of bark was COX-2 selective, while the leaves were COX-1 selective.

The results above are a good indication of a potential anti-inflammatory action of these extracts on the COX and 5-LOX system. The actual efficacy of an anti-inflammatory activity needs to be validated by testing the isolated bioactive compounds in a cell-based environment (*in vitro*) and in a biological system (*in vivo*). Additional studies are ongoing to isolate the bioactive compounds from the active fractions.

## 4. Conclusion

A combination of anti-inflammatory and antioxidant activities constitutes a good indication on potential anti-inflammatory activity of drug [39, 40]; keeping this in minds the ethanol extract of the plant warrants further studies by isolation of bioactive components. The pathway mechanism for the dual inhibition of the bioactive component against 5-LOX and COX needs to be investigated. The clinical significance of these data is quite clear that they support a role for *Canarium patentinervium *Miq. (Burseraceae Kunth.) as a source of lead compounds in the management of inflammatory diseases. 

## Figures and Tables

**Table 1:** Extraction yield and the total phenolic and flavonoid contents of the six extracts of *Canarium patentinervium* Miq.

Extracts	Extraction yield, % (w/w)	Total phenolic content, gallic acid equivalent (mg/g)	Total flavonoid content, quercetin equivalent (mg/g)
LH	1.25	6.61 ± 0.01	18.99 ± 0.02
LC	1.11	14.28 ± 0.03	12.57 ± 0.04
LE	6.45	204.97 ± 0.05	125.32 ± 0.03
BH	1.04	5.19 ± 0.01	1.55 ± 0.02
BC	0.40	13.99 ± 0.03	12.14 ± 0.02
BE	2.61	100.26 ± 0.01	24.57 ± 0.02

LH: leaf hexane extract, LC: leaf chloroform extract, LE: leaf ethanol extract, BH: bark hexane extract, BC: bark chloroform extract, and BE: bark ethanol extract.

**Table 2:** Antioxidant values of *Canarium patentinervium* Miq.

Extracts	ABTS assay, EC_50_ (*μ*g/mL)	FRAP assay, FRAP value (*μ*g/mL)	*β*-Carotene bleaching assay, EC_50_ (*μ*g/mL)	Superoxide dismutase assay, IC_50_ (*μ*g/mL)
LH	27.30 ± 0.01	2845.00 ± 0.15	9.39 ± 0.05	80.54 ± 0.03
LC	31.10 ± 0.02	1329.00 ± 0.18	21.81 ± 0.04	26.84 ± 0.03
LE	2.28 ± 0.01	200.00 ± 0.07	6.04 ± 0.02	3.05 ± 0.01
BH	521.00 ± 0.01	3531.00 ± 0.14	125.00 ± 0.08	238.00 ± 0.04
BC	34.90 ± 0.02	1242.00 ± 0.19	50.40 ± 0.03	20.13 ± 0.02
BE	0.93 ± 0.01a	67.00 ± 0.32	7.04 ± 0.04	8.51 ± 0.07
AA	1.54 ± 0.06	347.00 ± 0.23	NA	NA
QC	0.88 ± 0.03a	86.00 ± 0.24	1.64 ± 0.04b	NA
TRO	0.68 ± 0.02	33.00 ± 0.54	1.65 ± 0.03b	NA
SOD	NA	NA	NA	1.59 ± 0.04

LH: leaf hexane extract, LC: leaf chloroform extract, LE: leaf ethanol extract, BH: bark hexane extract, BC: bark chloroform extract, BE: bark ethanol extract, AA: ascorbic acid, QC: quercetin, TRO: trolox, and SOD: superoxide dismutase enzyme.

Data were obtained from three independent experiments, each performed in triplicates (*n* = 9) and represented as mean ± SD.

Values with the same letter are not significantly different (*P* < 0.05) according to Tukey, multiple comparison test.

**Table 3:** Anti-inflammatory values of *Canarium patentinervium *Miq.

Extracts	Anti-inflammatory assay 5-LOX, IC_50_ (*μ*g/mL)	COX-1 inhibition assay IC_50_ (*μ*g/mL)	COX-2 inhibition assay IC_50_ (*μ*g/mL)	COX-1/COX-2 ratio
LH	206.00 ± 0.02	>100	>100	NA
LC	104.69 ± 0.04	>100	>100	NA
LE	49.66 ± 0.02	0.60 ± 0.01	1.07 ± 0.01	0.56
BH	110.07 ± 0.04	>100	>100	NA
BC	29.53 ± 0.03	>100	>100	NA
BE	59.06 ± 0.07	11.41 ± 0.03	9.39 ± 0.03	1.22
NDGA	29.19 ± 0.02	NA	NA	NA
INDO	NA	0.29 ± 0.04	0.26 ± 0.03	1.11

LH: leaf hexane extract, LC: leaf chloroform extract, LE: leaf ethanol extract, BH: bark hexane extract, BC: bark chloroform extract, BE: bark ethanol extract, NDGA: nordihydroguaiaretic acid, and INDO: indomethacin.

Data were obtained from three independent experiments, each performed in triplicates (*n* = 9), and represented as mean ± SD.

Values with the same letter are not significantly different (*P* < 0.05) according to Tukey, multiple comparison test.
